# Experimental Systems in the Co‐Construction of Scientific Knowledge**

**DOI:** 10.1002/bewi.202200016

**Published:** 2022-09-09

**Authors:** Michel Morange

**Affiliations:** ^1^ Institut d'histoire et de philosophie des sciences et des techniques (IHPST) Paris

**Keywords:** epistemic things, experimental systems, metaphor, molecular biology, value of truth

## Abstract

The publication of *Toward a History of Epistemic Things* 25 years ago was a landmark in science studies. Not only was the book a brilliant overview of new research trends, but it was also a personal and highly original contribution because of its emphasis on the major role of experimental systems in the construction of scientific knowledge. The paths that it opened have not yet been fully explored. More seriously, the ambition of the author to reinforce the value of scientific knowledge by the role of experimental systems in its construction has not been pursued.

## 1 Introduction

Hans‐Jörg Rheinberger's publication of *Toward a History of Epistemic Things* 25 years ago marked the intersection of two different movements.^1^ The molecular revolution in the life sciences between 1940 and 1960 was followed by the development of genetic engineering, the characterization of genes implicated in human diseases and the sequencing of the human genome. Interest in science studies was profoundly renewed in the early 1960s and has since become an independent discipline critical of the historical narratives hitherto related. The sciences develop not along a steady pathway toward advances in knowledge, but follow an irregular path dependent on the social context and on historical vicissitudes. Instead of contributing to the great progressive discourses once in vogue, more recent historical narratives have concentrated on the way scientific knowledge has been constructed and on the controversies between groups of researchers who played a decisive role. The study of these disputes and their resolution moved the focus away from a naïve vision of scientific progress.

Rheinberger's book found its place perfectly at the crossroads of these two movements. It describes a little‐known episode in the history of molecular biology: the development of in vitro systems for the synthesis of proteins in Paul Zamecnik's laboratory in Boston. While the book greatly enriched the emerging more nuanced discourse, we will see how it truly transformed the field. The writing of this book also marked Rheinberger's departure from molecular biology research to science studies. My own conversion was much more gradual, but it started at about the same time. I was inspired by his articles that preceded the publication of his book. We soon forged a friendship that lasts to this day and of which this text is a new expression.

## 2 An Important Book

My aim is not to analyze Rheinberger's book again but to highlight its originality and thus to use it as a reference in examining the recent historiography of the life sciences. It is first of all a quite classical narrative of a neglected episode in the history of molecular biology. Indeed, it is not the study of a particular controversy. The social and institutional context is mentioned, but is not the main protagonist of the story. While not neglecting reference to laboratory notebooks, Rheinberger bases his account above all on the published articles. There is no trace of a frenetic search by the author for hitherto overlooked documents that would reveal the hidden strategies of scientists. Though he does not omit the theoretical dimension of the sciences, his scrutiny focuses mainly on the co‐evolution of the experimental system and the representations thereof given by the experimenters as well as the slow transformation of epistemic things into new scientific concepts.

The book offers its readers an extraordinary wealth of interpretations; Rheinberger introduces all the analytical categories that emerged in this new wave of science studies. But, far from being a catalog, the book is first and foremost a highly original approach to the scientific phenomenon, based on authors rarely mentioned, such as Jacques Derrida. In its numerous references to French authors, the book sometimes has an encyclopedic character. The author borrows approaches from textual studies as well as the history of art, and he does not hesitate to venture into the realm of metaphysics, addressing, for example, the question of “possibles.”

Some characteristics of the book are particularly worthy of attention. The first is its organization: the chapters describing scientific work alternate with those enabling analysis. Is this a reflection in the book's structure of the ongoing transition in the author's academic life or a stronger position statement according to which the scientific level of analysis must be clearly distinguished from the epistemological level, with the two disciplines nonetheless best dealt with together?

Much of the enduring interest in the book comes from the conclusion that the chosen experimental system is representative of all experimental systems. In his latest book, *Spalt und Fuge*, Rheinberger seeks to distinguish the different types of experimental systems. Unfortunately, I don't have access to this recent book as it has yet to be translated into English. The title of the 1997 book emphasized epistemic things and not experimental systems, yet it seems to me that the study of such systems is the most original aspect of the work. It is by the mediation of these experimental systems that we can reconcile a radically new vision of the construction of scientific knowledge and our confidence in the latter. Our scientific concepts are not arbitrary, the fruit of an a priori conception of phenomena, but are the result of a co‐construction imposed through the life of the experimental system by that which these concepts attempt to explain.

## 3 Provisional Assessment after 25 Years of Study

Have we exploited the potential offered by this book? A first, somewhat dispiriting remark is that two caveats firmly raised by Rheinberger have often been overlooked. First, explanations must not substitute a sociological determinism for a previously favored historical‐scientific determinism, and second it is necessary to retain scientific knowledge's particular value of truth. The work of deconstruction has sometimes prevailed over that of reconstruction. I shall take a single example with which I am familiar, even though it concerns the life sciences of the nineteenth century, that of Pasteur. Pasteur was an easy target for the new history of science; both in his life and work, myth often replaced fact. *The Private Science of Louis Pasteur* by Gerald Geison, which appeared just before *Epistemic Things*, exemplifies both the strengths as well as failings of many books published within the framework of this new history.^2^ Based largely on Pasteur's laboratory notebooks that were long inaccessible to researchers, it rectified numerous aspects of the myth, for instance revealing that Joseph Meister was not the first person to be vaccinated against rabies. Beyond these necessary corrections, the book added nothing to the understanding of Pasteur's scientific approach nor of the numerous scientists who renewed the study of micro‐organisms in the nineteenth century. Geison clearly shows that the canonical narratives of the invention of vaccines are inexact and that Pasteur deliberately concealed and even deformed certain aspects, but he does not give a precise description similar to those of Rheinberger of the progressive development of vaccines.

In the historiography of the life sciences in the twentieth century, I shall begin with Rheinberger's superb article on the German geneticist Alfred Kühn. In the 1930s, Kühn attempted, in parallel with other scientists, to understand the function of genes by using the moth *Ephestia* as a model system.^3^ This involved an extension of the notion of experimental systems to whole organisms. In the last three decades, numerous researchers have profitably trodden the same path, and today we have many studies of viruses, the fruit fly *Drosophila*, and the plant *Arabidopsis thaliana*. Angela Creager's studies on the introduction of radioactive markers into biology fall within the same movement, as their use engenders the development of new experimental systems.^4^

But the emergence in the life sciences of new disciplines—epigenetics, systems biology, and synthetic biology—and the growing role of computer science and databanks have given rise to numerous studies. The entirety of these phenomena and their societal issues have reinvested sociology with a certain pre‐eminence to the detriment of studies that are more limited in ambition, but more informative regarding the construction of scientific knowledge. Likewise, the exploitation of information from human genome sequencing has led to the emergence of so‐called personalized medicine, which focuses on the prevention of disease and the optimization of treatment. The highlighting of the economic, social, and ethical issues generated by this new medicine has taken precedence over the critical examination of knowledge meant to guide this new practice.

Lastly, a new approach to the sciences by philosophers has recently gained importance. The aim is no longer to distance oneself from the sciences and to examine the construction of knowledge critically, but to unite with the sciences and to participate in their development while injecting knowledge with a hint of philosophy. This movement is new in a discipline like immunology, but already longstanding in the biology of evolution.

Rheinberger's great merit was to replace experience, which allowed the construction of knowledge to be attributed to a single actor, with the experimental system. If there is a criticism to be leveled at his book, or rather a suggestion to be made, it would be to go beyond a purely rational description of the thought of scientists and to leave more room for images and metaphors,^5^ which almost always encumber their reflection. By their very nature, the latter obey multiple temporalities: a metaphor can be as old as humankind or conversely borrowed from a new technology. The articulation of these different temporalities engenders a complex dynamic that interacts with experimental systems to produce knowledge.

This rapid sketch of the recent evolution of historiography would not be complete without the acknowledgement of a sense of failure, doubtless shared by Rheinberger, regarding the scope of the work accomplished. Although the molecular revolution has become a standard lesson in all levels of schooling since *Epistemic Things*, one can only be greatly disappointed at the pitiful level of discussions on RNA vaccines.^6^ I still remember my surprise when, after having explained for over an hour to a journalist what these new vaccines consist of, I realized that he still thought that the antibodies produced after vaccination were directed against the RNA of the virus!

## 4 Conclusion

What purpose do science studies serve? My favored answer is that they provide principles of intelligibility, whence my great disillusion during recent events, and it seems to me, the need to rethink the objectives of our work. The more precise question perhaps would be, for whom is our work intended? Two diametrically opposed answers are possible. The first answer is that it is intended to help scientists better understand the very particular nature of their activity and to become more aware of what is at stake, as well as to help all citizens, because the personal and political choices they will have to make are often clarified by the scientific information they have at hand. The second answer is that it is intended only for specialists in the science studies, whose creation of new analytical categories will strengthen the autonomy of their discipline. Science studies form an independent discipline, but I am convinced that the work accomplished can and should benefit both scientists and the general public. Studies of science should open up to the outside world and not simply to other members of this small community. So much the better if there is great exchange with the sciences in certain fields. The creation of specific analytical categories is necessary, but care should be taken that these categories do not hamper interactions with other disciplines.

Twenty‐five years ago, Rheinberger initiated a new and original way of describing the scientific phenomenon. There remains much work ahead to take advantage of its full potential.

## Footnotes

n1 Rheinberger 1997.

n2 Geison 1995.

n3 Rheinberger 2000.

n4 Creager 2013.

n5 Reynolds 2018.

n6 De Chadarevian's contribution in this volume also deals with the paradox of diminishing public interest in and understanding of molecular biology at the time when molecular biology is starting to deliver on its promises.
